# Peripheral kynurenine/tryptophan ratio is not a reliable marker of systemic indoleamine 2,3-dioxygenase: A lesson drawn from patients on hemodialysis

**DOI:** 10.18632/oncotarget.15705

**Published:** 2017-02-25

**Authors:** Yuanhan Chen, Zhen Xie, Chenggen Xiao, Min Zhang, Zhilian Li, Jianteng Xie, Yusheng Zhang, Xingchen Zhao, Pengfei Zeng, Liyi Mo, Xinling Liang, Wei Shi

**Affiliations:** ^1^ Division of Nephrology, Guangdong General Hospital, Guangdong Academy of Medical Sciences, Guangzhou, China; ^2^ Department of Dermatology, Sichuan Academy of Medical Sciences & Sichuan Provincial People's Hospital, Chengdu, China; ^3^ Division of Nephrology, Xiangya Hospital, Central South University, Hunan, China; ^4^ Department of Gastroenterology, The Sixth Affiliated Hospital, Sun Yat-Sen University, Guangzhou, China; ^5^ Second Division of Internal Medicine, Wuhua People's Hospital, Guangdong, China; ^6^ Southern Medical University, Guangzhou, China; ^7^ Department of Nephrology, Dongguan People's Hospital, Guangdong Province, China

**Keywords:** indoleamine 2, 3-dioxygenase, kynurenine to tryptophan ratio, hemodialysis, immune tolerance, infection

## Abstract

Indoleamine 2,3-dioxygenase (IDO) has emerged as a pivotal enzyme for mediating immune tolerance. Because IDO metabolizes tryptophan into kynurenine, the plasma kynurenine/tryptophan (Kyn/Trp) ratio has been widely used as a marker of systemic IDO. Here, we evaluated the clinical value of using the plasma Kyn/Trp ratio to estimate cell-mediated immune responses to tuberculin skin testing and risk of new bacterial infection. We also compared the Kyn/Trp ratio to a novel IDO marker, the IDO median fluorescence index (MFI) of peripheral blood mononuclear cells, which was determined by flow cytometry. In 228 patients from two hemodialysis centers, the two IDO markers were higher in patients than in healthy controls but were not correlated with each other. *In vitro* experiments demonstrated that peripheral blood mononuclear cells could not metabolize tryptophan into kynurenine, indicating that the increased Kyn/Trp ratio was IDO-independent. Skin induration diameters of tuberculin skin testing were correlated with the IDO MFI (negatively), but not the Kyn/Trp ratio. Further, in a 24-month prospective cohort, the Kyn/Trp ratio was not correlated with clinical infection. Alternatively, patients with a higher IDO MFI had a lower accumulative infection-free survival rate. Using a Cox proportional hazard model, it was also revealed that a higher IDO MFI was significantly associated with new bacterial infection. Taken together, these results indicate that the Kyn/Trp ratio is not a reliable circulating IDO marker in hemodialysis patients. However, the IDO MFI reflects an immunocompromised state and thus might be a potential clinical marker of bacterial infection.

## INTRODUCTION

Indoleamine 2,3-dioxygenase (IDO), an intercellular rate-limiting enzyme that catalyzes the conversion of the essential amino acid tryptophan (Trp) into kynurenine (Kyn), has emerged as a pivotal molecule mediating immune tolerance [1]. Overactive IDO likely exerts toxicity in immune cells through multiple pathways. A Trp-depleted microenvironment can trigger the proliferative arrest, differentiation, and apoptosis of immune cells as well as disturbances in the energy metabolism of these cells [2, 3]. In addition, increased production of Kyn disrupts interleukin-2 signaling in memory CD4 T cells [4]. Moreover, IDO contains immunoreceptor tyrosine-based inhibitory motifs that participate in a positive feedback loop operating in a self-maintaining regulatory circuit [5]. In particular, IDO appears to be important in the generation and function of regulatory T (Treg) cells [6, 7].

Only a few specific subsets of cells appear to be capable of producing functional IDO. Dendritic cells (DCs) are the major source of peripheral IDO [8]. Approximately 3% of human immunodeficiency virus (HIV)-infected peripheral blood mononuclear cells (PBMCs) analyzed by flow cytometry, mainly plasmacytoid DCs, were IDO positive [9]. In addition to using flow cytometry to directly detect intercellular IDO, an indirect method was applied to detect IDO enzymatic activity *in vitro*. The latter method is based on the conversion of Trp to Kyn by IDO; high Trp-containing culture medium is applied to the cells, and IDO activity is estimated by determining Kyn levels or the Kyn/Trp ratio [10]. This method is used to reflect *in vivo* IDO activity by detecting the plasma Kyn/Trp ratio and has been increasingly employed in animal and clinical experiments [11–15].

An immunocompromised state and susceptibility to infection are the most profound features of uremia [16]. Infection is the second most common cause of death among hemodialysis patients [17] and imposes a heavy economic burden on these patients [18]. Recently, a Kyn/Trp ratio assay has been used to assess the immune state of hemodialysis patients [19]; however, the ratio remains to be verified as a marker for this purpose.

The clinical value of the ratio has been inconsistent. For example, one report demonstrated that it can predict kidney allograft rejection [20], but this finding could not be confirmed in a later study [21]. The roles of Trp and Kyn in infectious disease are also controversial. The depletion of Trp and enhancement of Kyn associated with IDO have been implicated in inhibiting the growth of certain bacteria and parasites. They also exhibit antiviral properties against several viruses, which is in contrast to the role of IDO in susceptibility to infection [22]. In animals and humans with renal failure, Trp levels are decreased, whereas Kyn levels are increased [23, 24], further raising the question of whether these changes signify increase peripheral IDO activity. Considering the low proportion of IDO-competent cells in the peripheral blood [9], it is unlikely that peripheral IDO influences plasma Trp and Kyn levels.

The present study focused on two representative hemodialysis centers of Guangdong General Hospital (GGH) and Wuhua People's Hospital (WHH) and aimed to investigate (1) the circulating IDO status in uremia, (2) the capacity of PBMCs to metabolize Trp, (3) whether the Kyn/Trp ratio reflects *in vivo* peripheral IDO activity, and (4) the relationship between circulating IDO and immune response and infection.

## RESULTS

### Circulating IDO in PBMCs increased in hemodialysis patients

**Figure 1:**
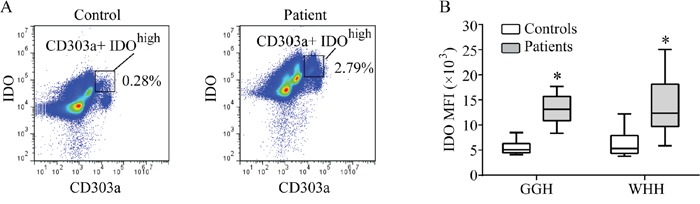
IDO expression in PBMCs **(A)** Typical blueprints of CD303a(+) IDO^high^ subsets in the controls and hemodialysis patients. Data were obtained by flow cytometry. **(B)** Difference in the IDO MFI of PBMCs between the controls and hemodialysis patients (*P<0.05, controls vs. patients). MFI: median fluorescence index; PBMCs: peripheral blood mononuclear cells.

No obvious subgroups of IDO-expressing PBMCs were identified in either healthy control subjects or hemodialysis patients from either GGH or WHH, and the proportion of CD303a (+) IDO^high^ cells was very low at less than 3% (Figure 1A). The median fluorescence index (MFI) was used to determine the peripheral IDO protein level. The IDO MFI in PBMCs was highly correlated with the IDO MFI in CD303a (+) cells (*r_s_*=0.718, P<0.001) across both groups. Thus, only the IDO MFI in PBMCs is reported in the remainder of this work. The IDO MFI was significantly higher in the hemodialysis patients from both hospitals compared to the healthy controls (Figure 1B).

### Plasma Kyn/Trp ratio was not correlated with circulating IDO in PBMCs

**Figure 2:**
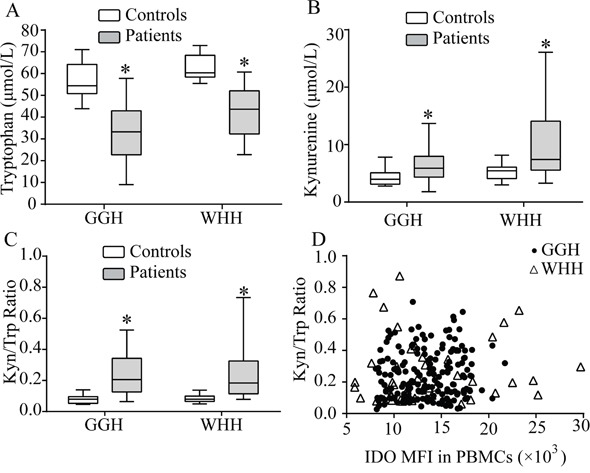
Relationships between peripheral blood Trp and its metabolites with IDO **(A)** Trp levels were significantly lower in the hemodialysis patients than in the controls. **(B)** Kyn levels were significantly higher in the hemodialysis patients than in the controls. **(C)** The Kyn/Trp ratio was significantly higher in the hemodialysis patients than in the controls. (*P<0.05, controls vs. patients). **(D)** The Kyn/Trp ratio did not show a correlation with the IDO MFI in PBMCs (GGH: *r_s_*0.059, P=0.426; WHH: *r_s_* 0.106, P=0.485). MFI: median fluorescence index; PBMCs: peripheral blood mononuclear cells.

The Trp level was significantly lower and the Kyn level and Kyn/Trp ratio were higher in hemodialysis patients from both GGH and WHH compared to the control subjects (Figures 2A-2C), in agreement with a previous report [19]. To reduce the potential influence of gender and age on IDO, Trp, and Kyn levels, the controls and patients were matched 1:2 based on gender and age (difference < 5 years). The results obtained after matching were consistent with the original analysis. Both the IDO MFI and the Kyn/Trp ratio were higher in the hemodialysis patients than in the controls (Supplementary Figure 1).

Although the IDO MFI and Kyn/Trp ratio were both elevated in hemodialysis patients, no correlation was observed between these variables (Figure 2D).

### PBMCs did not metabolize tryptophan *in vitro*

**Figure 3:**
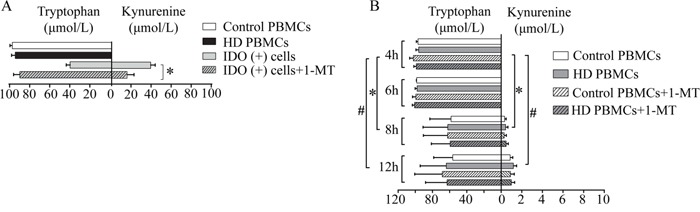
*In vitro* ability of PBMCs to metabolize Trp **(A)** Cells were cultured with 100 μmol/L Trp for 4 h. After culturing PBMCs isolated from the controls and the hemodialysis patients for 4 h, the Trp and Kyn levels in the culture medium did not change significantly. In 293 cells transfected with IDO(+), the concentration of Trp decreased and the Kyn level increased, but addition of the IDO-specific inhibitor 1-MT inhibited these changes (*P<0.05, IDO(+) cells vs. IDO(+) cells + 1-MT). **(B)** Co-culture of PBMCs and Trp for 12 h. At 4 and 6 h, generation of Kyn or loss of Trp was not observed, whereas at 8 and 12 h, Kyn generation of up to 2.0 μmol/L and a 40% decrease in Trp were observed. The addition of 1-MT did not influence the levels of Trp or Kyn. MFI: median fluorescence index; PBMCs: peripheral blood mononuclear cells.

Blood samples from the patients from GGH were analyzed to investigate the ability of PBMCs to metabolize Trp *in vitro*. Following the standard 4-h incubation with Trp in PBMCs [10], we did not detect any transformation of Trp to Kyn, in contrast to significant transformation in the positive control cells (HEK293T cells transfected with human IDO1) (Figure 3A).

When the incubation was extended to a maximum of 12 h, only a negligible transformation was observed after 8 h. In addition, this weak transformation was not inhibited by 1-methyl-D-tryptophan (1-MT) (Figure 3B). These results suggest that incubation with unstimulated PBMCs isolated from hemodialysis patients did not significantly reduce the level of Trp.

### IDO MFI, but not plasma Kyn/Trp ratio, potentially reflects delayed-type cell-mediated immune responses

**Figure 4:**
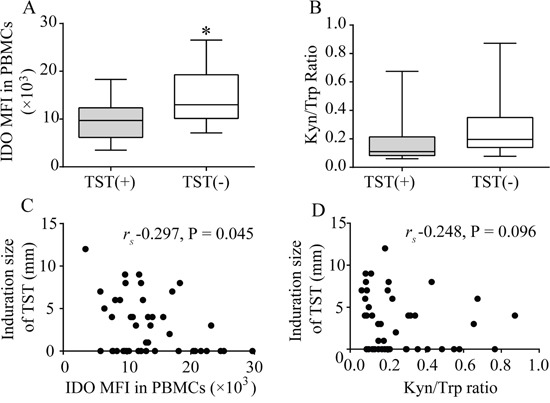
Relationship between IDO and tuberculin test results **(A)** The IDO MFI was higher for positive TST results than for negative TST results (*P<0.05). **(B)** The Kyn/Trp ratio did not differ significantly between negative and positive TST results. **(C)** The diameter of the TST induration was negatively correlated with the IDO MFI (*r_s_* -0.297, P=0.045). **(D)** The TST induration was not correlated with the Kyn/Trp ratio (*r_s_* -0.248, P=0.096). MFI: median fluorescence index; PBMCs: peripheral blood mononuclear cells; TST: tuberculin skin test.

Tuberculin skin testing (TST) was performed on the patients from WHH to evaluate delayed-type cell-mediated immune responses. In total, 10 of 46 hemodialysis patients and 9 of 10 healthy controls displayed positive TST results (indurations of more than 5 mm) (31.6% vs. 90.0%, P=0.003). The IDO MFI was higher in the patients with positive TST results than those with negative TST results (Figure 4A), but the Kyn/Trp ratio was approximately equal between groups (Figure 4B). The TST induration diameter was negatively correlated with the IDO MFI (Figure 4C) but was not correlated with the Kyn/Trp ratio (Figure 4D). Thus, the IDO MFI, but not the Kyn/Trp ratio, reflected a compromised cell-mediated immune status.

### Baseline IDO MFI, but not plasma Kyn/Trp ratio, was associated with infection-free survival

**Figure 5:**
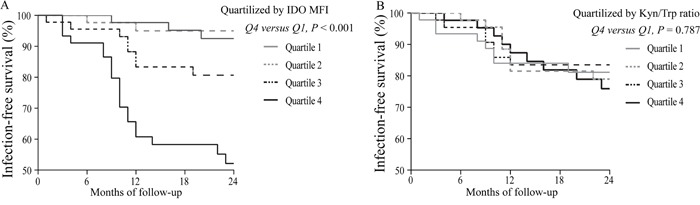
Infection-free survival curves based on the interquartile range of the IDO MFI or the Kyn/Trp ratio **(A)** Comparison of Q4 of the IDO MFI with the Q1 revealed a lower cumulative infection-free survival rate (P<0.01). **(B)** Comparison of the Q4 and Q1 of the Kyn/Trp ratio revealed no difference in the cumulative infection-free survival rate (P=0.787). MFI: median fluorescence index.

Among the GGH cohort, new bacterial infections (NBIs) were detected in 33 patients during the 24-month follow-up. The primary infections were 25 respiratory tract infections, 4 digestive tract infections, 3 cutaneous infections, and 1 long-term hemodialysis catheter-associated infection. The baseline IDO MFI and Kyn/Trp ratio were divided into quartiles from Q1 to Q4. Compared with Q1 of the IDO MFI, Q4 revealed a lower accumulative infection-free survival rate (Figure 5A). However, comparing Q4 and Q1 of the Kyn/Trp ratio revealed no difference in the accumulative infection-free survival rate (Figure 5B).

### Baseline IDO MFI, but not plasma Kyn/Trp ratio, was associated with NBI risk

Multivariate Cox regression analysis was performed by adjusting for age, baseline Geriatric Nutritional Risk Index (GNRI), months on dialysis, and quartiles of IDO MFI or Kyn/Trp ratio. A higher IDO MFI (Q4 vs. Q1) was associated with a significantly increased risk for NBI, whereas a higher Kyn/Trp ratio was not (Table 1).

**Table 1:** Relationships between IDO markers as well as other variables and new bacterial infection as determined by multivariate Cox regression analysis

Model 1 including IDO MFI		
Variables	Hazard ratio (95% CI)	P
IDO MFI (Quartile 4 vs. Quartile 1)	9.257 (2.723-31.475)	<0.001
Age (for every 10-year increase)	1.017 (0.807-1.281)	0.888
GNRI (for every 10-point increase)	1.284 (0.773-2.132)	0.335
Months on dialysis (for every 1-month increase)	1.001 (1.000-1.002)	0.045
**Model 2 including the Kyn/Trp ratio**		
**Variables**	**Hazard ratio (95% CI)**	**P**
Kyn/Trp ratio (Quartile 4 vs. Quartile 1)	0.634 (0.228-1.761)	0.382
Age (for every 10-year increase)	1.075 (0.842-1.371)	0.563
GNRI (for every 10-point increase)	1.056 (0.637-1.749)	0.834
Months on dialysis (for every 1-month increase)	1.001 (1.000-1.002)	0.059

Multivariate Cox regression analysis was performed by adjusting for 4 different variables. Models 1 and 2 were applied to investigate the quartiles of the two IDO markers, the IDO MFI and the Kyn/Trp ratio. In Model 1, high IDO MFI quartile (Quartile 4 vs. Quartile 1) was correlated with new bacterial infection, whereas in Model 2, high Kyn/Trp ratio quartile was not correlated with new bacterial infection. IDO: indoleamine 2,3-dioxygenase; MFI: median fluorescence index; GNRI: Geriatric Nutritional Risk Index.

## DISCUSSION

This study is the first investigation to validate the reliability of the Kyn/Trp ratio as a marker of peripheral IDO. PBMCs could not metabolize Trp into Kyn *in vitro*, indicating that systemic Trp metabolism is IDO-independent. The TST results suggested that the Kyn/Trp ratio does not reflect a cell-mediated immune response. Furthermore, during the 24 months of cohort observation, the increased baseline Kyn/Trp ratio was not related to a higher risk of NBI. These results do not support the widely used Kyn/Trp ratio as a marker of IDO. Alternatively, the IDO MFI was associated with inhibition of the immune response and NBI; therefore, we propose that this parameter is a promising a novel marker of IDO.

There are several explanations for the insufficiency of the Kyn/Trp ratio as a marker of IDO in the hemodialysis population. First, multiple mechanisms contribute to IDO-independent Trp loss, including diminished dietary Trp absorption, increased Trp elimination [25, 26], and Trp loss through hemodialysis [27]. Second, Trp can also be metabolized by L-tryptophan 2,3-dioxygenase (TDO), which increases when renal failure occurs [28, 29]. Third, inflammation and malnutrition are two significant characteristics of hemodialysis patients [29]. The proportion of Trp that is metabolized into Kyn will be enhanced in uremic malnutrition [30], whereas the synthesis of acute phase proteins during inflammation could consume Trp, such as in cancer patients experiencing an inflammatory response [31]. Finally, Trp is required for microbial growth, and its metabolite, Kyn, has short-term antimicrobial effects. This local enhanced innate immune mechanism could neutralize the immune tolerant role of circulating IDO in DCs. Because the above IDO-independent factors do not promote intercellular IDO expression, they also explain the clinical value of IDO MFI as a stable immunological marker for tuberculin reaction and NBI. However, our results cannot exclude the possibility that the Kyn/Trp ratio is a marker of other non-infectious conditions, such as malnutrition and cancer.

In addition to the Kyn/Trp ratio, an easily executed enzyme-linked immunosorbent assay has also been used to detect blood plasma IDO protein levels [32–34]. However, IDO is an intercellular enzyme, and there is no known secreted or extracellular form [35]. Our results did not show a correlation between plasma IDO level and IDO MFI (Supplementary Figure 2). Therefore, the significance of IDO protein levels in plasma was not explored in this study.

The results of TST, which was used to assess cell-mediated immunity in the patients from WHH, can be affected by nutritional factors. The patients at WHH had a higher GNRI nutritional score than those at GGH (Supplementary Table 1), which reduced the nutritional influence on the TST results. In addition, Bacille Calmette-Guerin (BCG) tuberculosis vaccination can also cause false-positive TST results within 15 years after vaccination [36, 37]. Because BCG vaccination was routinely used in newborns in the area local to WHH, the TST results in our study could be representative of adult immune status.

This study has some limitations. First, only baseline IDO markers were detected. Additionally, bacterial infection is a multi-factorial disease; due to the limited sample size, only age, nutrition, and dialysis vintage were adjusted in the multivariate model. Furthermore, immune disturbance may lead to non-bacterial infections such as those caused by viruses or fungi. Because our results were gathered from a specific hemodialysis population, they cannot be directly applied to other populations. Therefore, further in-depth investigations of the clinical significance of IDO markers are required.

In conclusion, our results showed that the Kyn/Trp ratio is not a reliable marker of IDO in hemodialysis patients. However, IDO MFI detected by flow cytometry does reflect immunocompromised status in these patients and might be useful as a marker of infection.

## MATERIALS AND METHODS

### Study design and rationale

The part of the study conducted at GGH was an observational, cross-sectional and prospective 24-month cohort study. One hundred and eighty-two patients on hemodialysis were included for the cross-sectional survey; all had been screened for blood-borne infectious diseases in December 2013. Ten healthy medical staff members were selected as the controls. After the cross-sectional survey (baseline), NBIs were prospectively recorded.

The part of the study conducted at WHH was a cross-sectional observational study that included 46 hemodialysis patients and 10 healthy controls.

Blood samples were obtained from the patients and controls after informed consent. The use of samples complied with the Declaration of Helsinki and was approved by the Ethics Committee of GGH and the department in charge of scientific research at WHH.

Basic clinical data for the patients from the two hospitals are shown in Supplementary Table 1. None of the enrolled patients exhibited evidence of infection or cancer at baseline. Additionally, they had not been hospitalized and had not undergone surgery or treatment with immunosuppressive or nutritional agents in the previous 2 months. No patients had active tuberculosis based on chest X-rays, symptoms of fever or cough, and body weight loss. The healthy volunteers had no infectious disease manifestations within 1 week before blood was drawn, and all underwent a health examination no longer than 6 months prior to beginning the study.

### Definition of infection/immune-relevant markers

The GNRI was applied as a nutritional assessment index [38]. Its value for nutritional assessments has been authenticated among non-geriatric individuals [39]. The GNRI is calculated using the following formula: GNRI=[14.89×serum albumin (g/dl)]+[41.7×(body weight/ideal body weight)]. We defined ideal body weight in the GNRI formula as the value corresponding to height and a BMI of 22, which has been shown to be valid in previous studies of Asian patients [38, 40, 41].

In the cohort study conducted at GGH, NBI was diagnosed by a physician superior to the attending doctors after comprehensive consideration of clinical symptoms, signs, bacterial culture, procalcitonin results, and reactions following the use of antibiotics.

TST was performed to evaluate cell-mediated immune responses using the standard Mantoux method. The patients at WHH were younger than those at GGH and generally consumed a high-protein diet; consequently, their malnutrition rate was low [42], which tended to reduce interference in immune evaluations. Thus, this part of the study was only conducted at WHH. In addition, because the uremic patients presented weakened immune responses and a 5-mm induration rather than the conventional 10-mm induration, which would increase TST sensitivity [43], a 5-mm induration was defined as the TST-positive cutoff.

### IDO assay

Flow cytometry was employed to directly detect IDO in PBMCs [9]. CD303a was used as a marker of peripheral plasmacytoid DCs. After isolation, PBMCs were washed twice and stained with FITC-conjugated anti-human CD303a (Clone 201A, eBioscience). After fixation and permeabilization with 4% formaldehyde and saponin buffer, respectively, the cells were stained with anti-IDO antibodies (0.5 mg/mL, Enzo Life Science) for 60 min and then with Alexa Fluor® 647-labeled donkey anti-rabbit antibodies (Invitrogen, Carlsbad, CA, USA). Fluorescence was analyzed by flow cytometry using an AccuriC6 instrument (BD Biosciences, Oxford, UK), and the MFI was measured post-acquisition using FlowJo (Tree Star, Inc.).

Trp and Kyn concentrations in plasma or culture medium supernatants were simultaneously measured using a high-performance liquid chromatography (HPLC) system (Shimadzu LC-3A, Tokyo, Japan) according to our previously described method [24]. Briefly, a 20-μL sample of deproteinized plasma or supernatant was injected into a Sinochrom ODS-BP C18 column (150 mm × 4.6 mm, 4.5 μm or 5.0 μm). The spectrophotometer was set to 225 nm to detect Trp and Kyn. The mobile phase of the system was composed of 15 mmol/L sodium acetate solution containing 5% (*v/v*) acetonitrile (pH 4.8) and was maintained at a flow rate of 1.0 mL/min. The detection limits were 0.8 μmol/L for Trp and 0.08 μmol/L for Kyn [24].

To determine the ability of IDO to catalyze the conversion of Trp to Kyn in circulating PBMCs, IDO enzymatic activity in PBMCs was measured *in vitro* as previously described [10]. Briefly, fresh PBMCs were washed twice and resuspended in Hanks buffer containing 100 μM tryptophan (Sigma–Aldrich, St. Louis, MO, USA) and incubated for 4 h. The supernatants were harvested and assayed to measure the concentrations of Kyn (used as a surrogate for IDO activity) and Trp. IDO was transfected into HEK293T cells, which were used as positive control cells for Trp metabolism. Human IDO1 cDNA was inserted into the mammalian expression vector pCMV-3×Flag, and the constructs were used for transfection with Lipofectamine 2000 according to the manufacturer's instructions (Invitrogen). IDO1 overexpression in HEK293 cells was verified by western blotting (data not shown). To exclude the possibility of delayed transformation from Trp to Kyn, the incubation was extended to 6 h, 8 h and 12 h. The incubation time span for Trp was not extended because the MTT test results had already shown a disturbance of energy metabolism in PBMCs after 8 h of incubation (Supplementary Figure 3). To determine if this transformation was dependent on IDO, 1-MT (Sigma–Aldrich), an inhibitor of IDO enzymatic activity, was added to the PBMC suspensions immediately after washing.

### Statistical analyses

Data are presented as the means ± standard deviations (SDs) or medians (25th and 75th percentiles) and were analyzed using SPSS version 19.0 (Chicago, IL, USA). Differences between groups were compared using Pearson's χ^2^ test or the independent sample Mann–Whitney U test. Multiple results derived from different time points were assessed by repeated-measures analysis. Spearman's correlation coefficient (*r_s_*) was used to assess the correlation between two variables. Infection-free survival rates were calculated using the Kaplan-Meier method and compared using the log rank test. Cox proportional hazard regression analysis was used to identify risk factors for future infection. The relationship between peripheral IDO level and future infection was evaluated by Cox regression analysis based on monthly kinetic modeling as a time-dependent covariate. Two-tailed tests were conducted for all comparisons, and P<0.05 was considered significant.

## SUPPLEMENTARY MATERIALS FIGURES AND TABLES



## Abbreviations

IDO: Indoleamine 2,3-dioxygenase
Trp: tryptophan
Kyn: kynurenine
MFI: median fluorescence index
NBI: new bacterial infection
TST: tuberculin skin testing
DCs: dendritic cells
HIV: human immunodeficiency virus
PBMCs: peripheral blood mononuclear cells
GGH: Guangdong General Hospital
WHH: Wuhua People's Hospital
GNRI: Geriatric Nutritional Risk Index
HPLC: high-performance liquid chromatography
1-MT: 1-methyl-D-tryptophan
BCG: Bacille Calmette-Guerin

## References

[1] Medzhitov R, Shevach EM, Trinchieri G, Mellor AL, Munn DH, Gordon S, Libby P, Hansson GK, Shortman K, Dong C, Gabrilovich D, Gabryšová L, Howes A (2011). Highlights of 10 years of immunology in Nature Reviews Immunology. Nat Rev Immunol.

[2] Munn DH, Sharma MD, Baban B, Harding HP, Zhang Y, Ron D, Mellor AL (2005). GCN2 kinase in T cells mediates proliferative arrest and anergy induction in response to indoleamine 2,3-dioxygenase. Immunity.

[3] Eleftheriadis T, Pissas G, Antoniadi G, Spanoulis A, Liakopoulos V, Stefanidis I (2014). Indoleamine 2,3-dioxygenase increases p53 levels in alloreactive human T cells, and both indoleamine 2,3-dioxygenase and p53 suppress glucose uptake, glycolysis and proliferation. Int Immunol.

[4] Dagenais-Lussier X, Aounallah M, Mehraj V, El-Far M, Tremblay C, Sekaly RP, Routy JP, van Grevenynghe J (2016). Kynurenine reduces memory CD4 T-cell survival by interfering with interleukin-2 signaling early during HIV-1 infection. J Virol.

[5] Pallotta MT, Orabona C, Volpi C, Vacca C, Belladonna ML, Bianchi R, Servillo G, Brunacci C, Calvitti M, Bicciato S, Mazza EM, Boon L, Grassi F (2011). Indoleamine 2,3-dioxygenase is a signaling protein in long-term tolerance by dendritic cells. Nat Immunol.

[6] Puccetti P, Grohmann U (2007). IDO and regulatory T cells: a role for reverse signalling and non-canonical NF-kappaB activation. Nat Rev Immunol.

[7] Mezrich JD, Fechner JH, Zhang X, Johnson BP, Burlingham WJ, Bradfield CA (2010). An interaction between kynurenine and the aryl hydrocarbon receptor can generate regulatory T cells. J Immunol.

[8] Munn DH, Sharma MD, Lee JR, Jhaver KG, Johnson TS, Keskin DB, Marshall B, Chandler P, Antonia SJ, Burgess R, Slingluff CL, Mellor AL (2002). Potential regulatory function of human dendritic cells expressing indoleamine 2,3-dioxygenase. Science.

[9] Boasso A, Herbeuval JP, Hardy AW, Anderson SA, Dolan MJ, Fuchs D, Shearer GM (2007). HIV inhibits CD4+ T-cell proliferation by inducing indoleamine 2,3-dioxygenase in plasmacytoid dendritic cells. Blood.

[10] Braun D, Longman RS, Albert ML (2005). A two-step induction of indoleamine 2,3 dioxygenase (IDO) activity during dendritic-cell maturation. Blood.

[11] Schröcksnadel K, Wirleitner B, Winkler C, Fuchs D (2006). Monitoring tryptophan metabolism in chronic immune activation. Clin Chim Acta.

[12] Feder-Mengus C, Wyler S, Hudolin T, Ruszat R, Bubendorf L, Chiarugi A, Pittelli M, Weber WP, Bachmann A, Gasser TC, Sulser T, Heberer M, Spagnoli GC (2008). High expression of indoleamine 2,3-dioxygenase gene in prostate cancer. Eur J Cancer.

[13] O’Connor JC, André C, Wang Y, Lawson MA, Szegedi SS, Lestage J, Castanon N, Kelley KW, Dantzer R (2009). Interferon-gamma and tumor necrosis factor-alpha mediate the upregulation of indoleamine 2,3-dioxygenase and the induction of depressive-like behavior in mice in response to bacillus Calmette-Guerin. J Neurosci.

[14] Maneechotesuwan K, Wongkajornsilp A, Adcock IM, Barnes PJ (2015). Simvastatin suppresses airway IL-17 and upregulates IL-10 in patients with stable COPD. Chest.

[15] Ploder M, Spittler A, Schroecksnadel K, Neurauter G, Pelinka LE, Roth E, Fuchs D (2009). Tryptophan degradation in multiple trauma patients: survivors compared with non-survivors. Clin Sci (Lond).

[16] Betjes MG (2013). Immune cell dysfunction and inflammation in end-stage renal disease. Nat Rev Nephrol.

[17] Allon M, Depner TA, Radeva M, Bailey J, Beddhu S, Butterly D, Coyne DW, Gassman JJ, Kaufman AM, Kaysen GA, Lewis JA, Schwab SJ, HEMO Study Group (2003). Impact of dialysis dose and membrane on infection-related hospitalization and death: results of the HEMO Study. J Am Soc Nephrol.

[18] Berman SJ, Johnson EW, Nakatsu C, Alkan M, Chen R, LeDuc J (2004). Burden of infection in patients with end-stage renal disease requiring long-term dialysis. Clin Infect Dis.

[19] Schefold JC, Zeden JP, Fotopoulou C, von Haehling S, Pschowski R, Hasper D, Volk HD, Schuett C, Reinke P (2009). Increased indoleamine 2,3-dioxygenase (IDO) activity and elevated serum levels of tryptophan catabolites in patients with chronic kidney disease: a possible link between chronic inflammation and uraemic symptoms. Nephrol Dial Transplant.

[20] Brandacher G, Cakar F, Winkler C, Schneeberger S, Obrist P, Bösmüller C, Werner-Felmayer G, Werner ER, Bonatti H, Margreiter R, Fuchs D (2007). Non-invasive monitoring of kidney allograft rejection through IDO metabolism evaluation. Kidney Int.

[21] Lahdou I, Sadeghi M, Daniel V, Schenk M, Renner F, Weimer R, Löb S, Schmidt J, Mehrabi A, Schnitzler P, Königsrainer A, Döhler B, Opelz G, Terness P (2010). Increased pretransplantation plasma kynurenine levels do not protect from but predict acute kidney allograft rejection. Hum Immunol.

[22] Mehraj V, Routy JP (2015). Tryptophan Catabolism in Chronic Viral Infections: Handling Uninvited Guests. Int J Tryptophan Res.

[23] Pawlak D, Tankiewicz A, Mysliwiec P, Buczko W (2002). Tryptophan metabolism via the kynurenine pathway in experimental chronic renal failure. Nephron.

[24] Xiao C, Chen Y, Liang X, Xie Z, Zhang M, Li R, Li Z, Fu X, Yu X, Shi W (2014). A modified HPLC method improves the simultaneous determination of plasma kynurenine and tryptophan concentrations in patients following maintenance hemodialysis. Exp Ther Med.

[25] Holmes EW, Kahn SE (1987). Tryptophan distribution and metabolism in experimental chronic renal insufficiency. Exp Mol Pathol.

[26] Tsubakihara Y, Takabatake Y, Oka K, Shoji T, Togawa M, Okada N, Takahito I, Imai E (2003). Effects of the oral adsorbent AST-120 on tryptophan metabolism in uremic patients. Am J Kidney Dis.

[27] Unge G, Lins LE, Hultman E (1977). Tryptophan in patients on chronic haemodialysis. Lancet.

[28] Pawlak D, Tankiewicz A, Matys T, Buczko W (2003). Peripheral distribution of kynurenine metabolites and activity of kynurenine pathway enzymes in renal failure. J Physiol Pharmacol.

[29] Saito K, Fujigaki S, Heyes MP, Shibata K, Takemura M, Fujii H, Wada H, Noma A, Seishima M (2000). Mechanism of increases in L-kynurenine and quinolinic acid in renal insufficiency. Am J Physiol Renal Physiol.

[30] Le FN, Otten W, Merlot E (2011). Tryptophan metabolism, from nutrition to potential therapeutic applications. Amino Acids.

[31] Preston T, Slater C, McMillan DC, Falconer JS, Shenkin A, Fearon KC (1998). Fibrinogen synthesis is elevated in fasting cancer patients with an acute phase response. J Nutr.

[32] Eleftheriadis T, Liakopoulos V, Antoniadi G, Stefanidis I, Galaktidou G (2011). Indoleamine 2,3-dioxygenase is increased in hemodialysis patients and affects immune response to hepatitis B vaccination. Vaccine.

[33] Eleftheriadis T, Yiannaki E, Antoniadi G, Liakopoulos V, Pissas G, Galaktidou G, Stefanidis I (2012). Plasma indoleamine 2,3-dioxygenase and arginase type I may contribute to decreased blood T-cell count in hemodialysis patients. Ren Fail.

[34] Eleftheriadis T, Antoniadi G, Liakopoulos V, Stefanidis I, Galaktidou G (2012). Plasma indoleamine 2,3-dioxygenase concentration is increased in hemodialysis patients and may contribute to the pathogenesis of coronary heart disease. Ren Fail.

[35] Mellor AL, Munn DH (2004). IDO expression by dendritic cells: tolerance and tryptophan catabolism. Nat Rev Immunol.

[36] Wang L, Turner MO, Elwood RK, Schulzer M, FitzGerald JM (2002). A meta-analysis of the effect of Bacille Calmette Guerin vaccination on tuberculin skin test measurements. Thorax.

[37] Lee SS, Chou KJ, Dou HY, Huang TS, Ni YY, Fang HC, Tsai HC, Sy CL, Chen JK, Wu KS, Wang YH, Lin HH, Chen YS (2010). High prevalence of latent tuberculosis infection in dialysis patients using the interferon-gamma release assay and tuberculin skin test. Clin J Am Soc Nephrol.

[38] Bouillanne O, Morineau G, Dupont C, Coulombel I, Vincent JP, Nicolis I, Benazeth S, Cynober L, Aussel C (2005). Geriatric Nutritional Risk Index: a new index for evaluating at-risk elderly medical patients. Am J Clin Nutr.

[39] Naber TH, de Bree A, Schermer TR, Bakkeren J, Bär B, de Wild G, Katan MB (1997). Specificity of indexes of malnutrition when applied to apparently healthy people: the effect of age. Am J Clin Nutr.

[40] Tsai MT, Hu FH, Lien TJ, Chen PJ, Huang TP, Tarng DC (2014). Interaction between geriatric nutritional risk index and decoy receptor 3 predicts mortality in chronic hemodialysis patients. Am J Nephrol.

[41] Kobayashi I, Ishimura E, Kato Y, Okuno S, Yamamoto T, Yamakawa T, Mori K, Inaba M, Nishizawa Y (2010). Geriatric Nutritional Risk Index, a simplified nutritional screening index, is a significant predictor of mortality in chronic dialysis patients. Nephrol Dial Transplant.

[42] Chen Y, Li Z, Liang X, Zhang M, Zhang Y, Xu L, Zhong L, Shi W (2015). Effect of individual health education on hyperphosphatemia in the Hakkas residential area. Ren Fail.

[43] Richeldi L (2006). An update on the diagnosis of tuberculosis infection. Am J Respir Crit Care Med.

