# Supplementary material for: Peripheral kynurenine/tryptophan ratio is not a reliable marker of systemic indoleamine 2,3-dioxygenase: A lesson drawn from patients on hemodialysis
Source: Oncotarget. 2017 Feb 25;8(15):25261–9. doi: 10.18632/oncotarget.15705 (PMC5421927; doi:10.18632/oncotarget.15705)
Supplement: Supplementary file 1 [file oncotarget-08-25261-s001.pdf]

Peripheral kynurenine/tryptophan ratio is not a reliable marker of systemic indoleamine 2,3-dioxygenase: A lesson drawn from patients on hemodialysis

SUPPLEMENTARY MATERIALS

SUPPLEMENTARY FIGURES AND TABLE

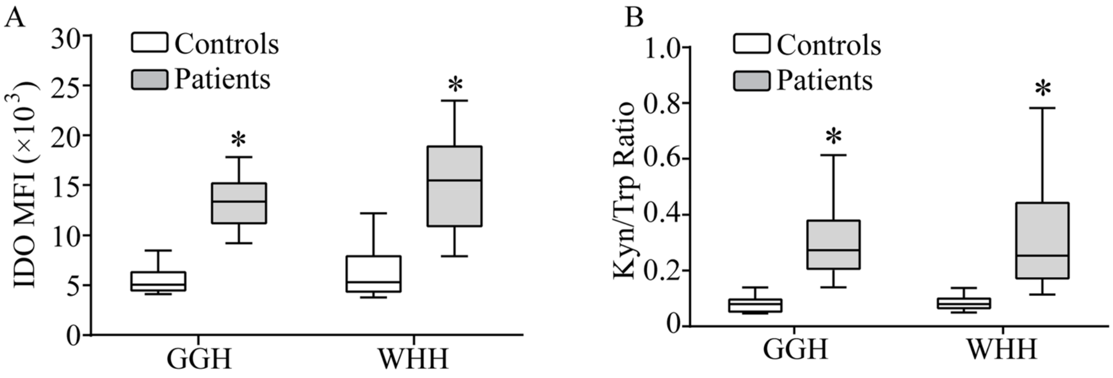

Supplementary Figure 1: Differences in IDO markers between the controls and the matched hemodialysis patients. The controls (n=10) and the patients (n=20) were matched 1:2 based on gender and age (difference <5 years). **(A)** The IDO MFI of the patients was significantly higher than that of the controls. **(B)** The Kyn/Trp ratio of the patients was significantly higher than that of the controls. *P<0.05 vs. the controls. GGH, Guangdong General Hospital; WHH, Wuhua People's Hospital; MFI: median fluorescence index.

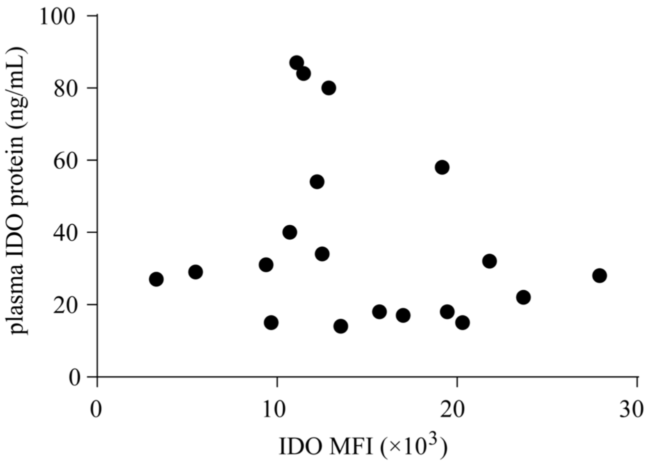

Supplementary Figure 2: Association between plasma IDO level and the IDO MFI. Methods: Whole peripheral blood samples were collected from 15 hemodialysis patients and 5 healthy controls. After centrifugation, PBMCs were isolated by Ficoll-Paque density centrifugation (GE Healthcare, Uppsala, Sweden), and the IDO MFI was detected. IDO protein level was detected using an anti-human IDO1 enzyme-linked immunosorbent assay (USCN Life Science, Wuhan, China). Results: Plasma IDO protein levels and the IDO MFI in PBMCs did not show a correlation (r_s= 0.255, P=0.277). MFI: median fluorescence index; PBMCs: peripheral blood mononuclear cells.

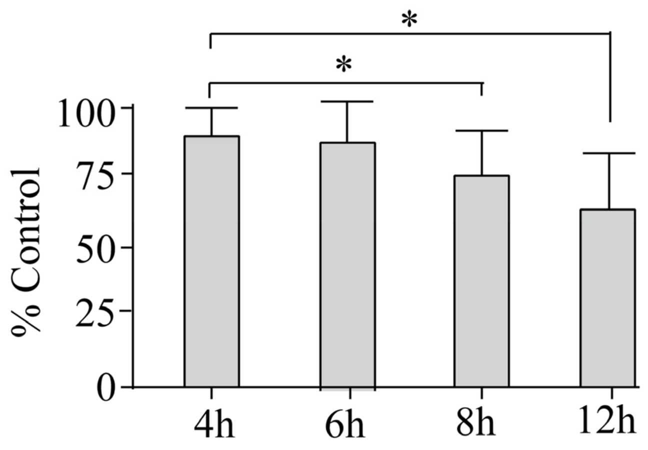

Supplementary Figure 3: Energy metabolism of PBMCs incubated with Tyr. Data were obtained using the MTT test. Method: Fresh PBMCs were washed twice, resuspended in Hanks buffer containing 100 µM tryptophan (Sigma–Aldrich, St Louis, MO, USA), and incubated for 4 h, 6 h, 8 h and 12 h. Cell energy metabolism was detected using an MTT assay kit (Nanjing KeyGen Biotech. Co. Ltd., China) according to the manufacturer's instructions. Results: After 8 and 12 h of *in vitro* co-incubation in buffer without serum and culture media, the metabolism of the PBMCs was noticeably decreased. A repeated-measures analysis was performed to assess the differences between the different time points: *$P<0.05$, results of the MTT test vs. those obtained at 4 h (control).

Supplementary Table 1: Clinical characteristics of hemodialysis patients in the two participating hospitals

Characteristics	GGH (n=182)	WHH (n=46)
Age, years	59±15	46±11
Male, n (%)	108 (59.3)	29 (63.0)
Dialysis vintage, months	40 (20,61)	36 (24, 59)
Diabetes, n (%)	43 (23.6)	5 (10.9)
Kt/V	1.60 (1.38, 1.80)	1.11 (0.98, 1.39)
Albumin, g/dL	3.19±0.31	3.60±0.34
iPTH, pg/mL	204 (103, 408)	351 (153, 768)
Ca×P, mg^2/dL^2	54.8 (44.0, 66.0)	73.9 (59.5, 90.0)
C-reactive protein, mg/L	3.1 (1.8, 6.6)	5.0 (3.0, 8.5)
Cholesterol, mmol/L	4.0 (3.3, 4.9)	5.0 (3.0, 9.3)
GNRI	89.4±7.1	96.3±9.5

GGH is the Professional Blood Purification Quality Administration and Control Center of Guangdong Province and represents an urban academic hospital in developed areas of China. WHH is a county hospital in an under-developed agricultural area of Guangdong Province. GGH: Guangdong General Hospital; WHH: Wuhua People's Hospital; iPTH: intact parathyroid hormone; Ca×P: product of calcium and phosphorus; GNRI: Geriatric Nutritional Risk Index.
